# Training tomorrow’s doctors: the impact of a Student Selected Component in Global Health during medical school

**DOI:** 10.15694/mep.2021.000150.1

**Published:** 2021-06-02

**Authors:** Natasha Matthews, Richard Walker

**Affiliations:** 1 Newcastle University

**Keywords:** Global health, Medical education, Curriculum planning, Career choice, Professional development

## Abstract

This article was migrated. The article was marked as recommended.

**Introduction:**Several reports highlight the importance of global health education (GHE) for training tomorrow’s doctors. In 2006, Newcastle University Medical School developed a Student Selected Component (SSC) in Global Health. We followed up students who completed the SSC to assess the impact on their experience as practising clinicians and postgraduate career development.

**Methods:**We developed an electronic survey including questions about speciality choice, postgraduate qualifications, extracurricular activity and international work. Surveys were sent to 72 SSC participants identified between 2006-2017 through the Newcastle University Alumni and Supporters network and social media.

**Results:** Surveys were returned by 37 (51%) SSC participants; 25 (71%) and 16 (46%) believed the SSC had influenced their clinical practice and career choice, respectively. Twenty-two (59%) obtained an intercalated degree programme, of whom nine (24%) did a Masters programme specifically in Global Health and four (11%), and two (5%) completed a Masters degree in Epidemiology and Control of Infectious Diseases respectively, both key themes within GHE. Four (11%) undertook, and 10 (29%) were considering postgraduate study related to global health, of whom three (9%) specified undertaking a Diploma in Tropical Medicine and Hygiene (DTM&H) and one (3%) studying a Masters degree in Public Health. Five (14%) had, and 19 (54%) were planning to work abroad, most referring to work in humanitarian or low resource settings and GHE programmes.

**Discussion and Conclusion:**Participation in an SSC in Global Health may affect positive change in students’ clinical practice and help inform academic and clinical career choice. Whilst a causative relationship cannot be inferred, the experience may support or increase the pursuit of additional global health-related qualifications, research and international health work. Medical schools that endeavour to produce graduates motivated to tackle our society’s global health challenges should champion comprehensive global health modules for students.

## Introduction

It is well documented by health professionals, medical students and expert panels that global health is an essential part of compulsory modern medical education ( Haq *et al.,* 2000; Bateman *et al.,* 2001; McAlister and Orr, 2006; Drain *et al.,* 2007; Houpt, Pearson and Hall, 2007; Jessop and Johnson, 2009; Tissingh, 2009; Frenk *et al.,* 2010; Rowson *et al.,* 2012; Willott *et al.,* 2012). The UK government also has an ongoing global health strategy recognising the need to engage in global health in our globalised world, not least to support the National Health Service (NHS) and secure good health for people in the UK ( Donaldson and Banatvala, 2007; Public Health England., 2014). Despite this, the development of standardised global health education (GHE) in UK medical schools has been arguably a slow process. In 2001, Newcastle University had no dedicated teaching on international health issues, and a survey of medical students showed 56% were dissatisfied with the amount of global health teaching and 61% would welcome more in both the core curriculum and as a special module ( Edwards, Rowson and Piachaud, 2001). Following a student’s request, a Student Selected Component (SSC) in Global Health was established in 2006 and has since become increasingly popular ( Dotchin, van den Ende and Walker, 2010). Students have continued to lobby for more global health in the compulsory curriculum, thus leading to increased formal global health teaching for all medical students ( Dotchin, van den Ende and Walker, 2010). This trend has continued with Newcastle Medical School implementing a reformed global health curriculum in 2018 ( Deivanayagam *et al.,* 2018) and running an intercalated Master of Research (MRes) programme in Global Health. Evidence shows such growth in GHE occurring nationally, with a 2015 study of UK medical schools finding an increase in the number of institutions reporting GHE in the core curriculum from 24% in 2006 (4/17 according to students) ( Dotchin, van den Ende and Walker, 2010) to 83% (according to medical school faculty) or 67% (according to students) and almost all offered optional global health programmes ( Matthews, Davies and Ward, 2020).

Since the development of the SSC, Newcastle University students in their fourth year (of five years) of medical school may undertake a six-week Global Health module covering weekly topics: Introduction to the epidemiology of major communicable diseases across the world, Non-communicable diseases and health systems, Climate change, aid and the philosophy of global health, Global maternal and child health, Global health policy, partnerships and governance and Migrant and refugee health and international health placements. Participants engage in a variety of lectures, seminars and clinic attendances, and a debate on relevant global health issues. Students also prepare presentations on topics pertinent to the week and are examined with a final presentation on a global health topic of their choice.

Although several works have argued the importance of GHE for training tomorrow’s doctors, to date, there is limited literature investigating the impact of GHE on student’s postgraduate career development. Thus, we seek to follow up students who have undertaken the Newcastle University medical school SSC in Global Health between 2006-2017 regarding how the SSC has impacted their experience as practising clinicians and postgraduate career development.

## Methods

### Survey design

We developed an electronic survey targeted at Newcastle Medical School alumni who have undertaken the SSC in Global Health using SurveyMonkey, an online survey development platform.

Survey questions were informed by the study of participants on the University of Wisconsin International Health Fellowship Program (IHFP) ( Ramsey *et al.,* 2004), identified by MEDLINE searches, including the terms ‘global health’ and ‘career influence’, as well as the survey of GHE in UK medical schools ( Matthews, Davies and Ward, 2020). The survey included questions about advanced training and speciality choice, extracurricular activity, postgraduate qualifications, international work, and the Global Health SSC’s influence on their clinical practice and careers.

### Administration of survey

We identified past participants in the Global Health SSC from records held by the current course administrator dating back to 2011. Before 2010, no data were retained by the University, and no hard copies were available. The first student to undertake the SSC in 2006 was known to the faculty due to their involvement in the development of the SSC ( Dotchin, van den Ende and Walker, 2010). Thus, a total of 74 past SSC participants were identified from 2006 and between 2011-2017. Current Newcastle University medical students who had undertaken the SSC in 2018 and 2019 were excluded from the data set as they had not yet begun their careers.

We gathered survey data between May and August 2019. Alumni who had not opted out of receiving communications were initially contacted with the survey through the Newcastle University Alumni and Supporters network on the 31st May and 18th June 2019. Following this, only 16 alumni completed the survey; therefore, we decided to extend the survey deadline and share it through social media. Where possible, NRM sent the survey to SSC participants via the social media service Facebook.Eight alumni, for whom a profile could not be found on Facebook, were contacted through NHS email. Contact details for two participants could not be located through Facebook, NHS email or the professional networking service, LinkedIn. Another two alumni responded that they had not completed the Global Health SSC during their undergraduate studies and were excluded from the study. All communication regarding the survey included a copy of the cover letter and a link to the survey.

### Analysis of survey responses

Descriptive statistics were calculated for quantitative analysis of the data. Thematic analysis was conducted on free-text responses to the questions “Describe the most significant influence of the Newcastle medical school SSC in Global Health on your clinical practice” and “Describe the most significant influence of the Newcastle medical school SSC in Global Health on your career choice”. NRM examined the data for common themes and combined them to realise a meaningful interpretation.

## Results/Analysis

Thirty-seven (51%) of 72 students who had undertaken the Global Health SSC responded to the survey within the time frame. These participants completed the SSC between 2011-2017 and graduated between 2013-2019 **( Table 1).** Of 16 respondents who completed the SSC in 2017 during the fourth year of medical school, 10 subsequently undertook an intercalated degree year (on top of their five-year medical degree), therefore graduated in 2019.

**Table 1:** Survey respondent characteristics

Characteristic	Previous SSC participants (n=37) n (%)
Age range, years (mean)	23-31 (26)
Gender	Male: 10 (27) Female: 27 (73)
Year completed SSC (mode)	2011-2017 (2017)
Year graduated (mode)	2013-2019 (2018/2019)
Employment status	To commence Foundation Programme August 2019: 11 (30) Employed, working full-time: 22 (59) Locum: 3 (8) Not employed, not looking for work: 2 (5)
Stage of Career	To commence Foundation Programme August 2019: 11 (30) Foundation Programme: 12 (32) Speciality and GP training: 10 (27) Not in training: 4 (11)

### Speciality Choice

The speciality choices of previous SSC participants were compared with those of UK medical graduates of 2015, one year after graduation ( Lambert, Smith and Goldacre, 2018), an age group similar to that of the study participants. Of previous SSC participants, one (3%) indicated Public Health as their top choice of speciality, compared with 28 (1%) of UK medical graduates, showing Public Health is one of the least popular choices of speciality amongst both groups **( Table 2).** Despite this, 16 (46%) of 35 respondents reported having considered applying to Public Health speciality training. The most popular choice of speciality was General Practice, with 27% of previous SSC participants and 28% of UK medical graduates selecting it as their top choice. The study also found that 11% of past SSC participants versus 1% of UK medical graduates were not interested in pursuing speciality or general practice training at all.

**Table 2:** Career speciality choices of previous SSC participants compared with UK medical graduates of 2015 one?year after graduation

Speciality	Previous SSC participants (n=37) n (%)	UK medical graduates of 2015 * n=3040 n (%)
Anaesthetics	2 (5)	480 (16)
Emergency Medicine	1 (3)	171 (6)
General Practice	10 (27)	844 (28)
Medicine	4 (11)	826 (27) Hospital medical specialities + Other medical specialities
Obstetrics and Gynaecology	3 (8)	164 (5)
Paediatrics and Child Health	5 (14)	261 (9)
Pathology	0	97 (3)
Psychiatry	1 (3)	165 (5)
Public Health	1 (3)	28 (1)
Radiology	2 (5)	92 (3)
Surgery	3 (8)	492 (16)
Other	1 (3) Tropical Medicine and Public Health	87 (3) Radiotherapy and oncology + Community health
Not pursuing speciality or general practice training	4 (11)	44 (1)
Not stated	0	105 (3)

* 2015 data ( Lambert, Smith and Goldacre, 2018)

Only one participant reported undertaking a rotation in Public Health during their two-year Foundation Programme. Three participants had accepted an Academic Foundation Programme (AFP) training post, two of whom stated their research project would be related to global health.

### Further Global Health Education

Twenty-two (59%) previous SSC participants had undertaken an intercalated degree programme. Table 3 highlights respondents’ degree choices, with nine (24%) reporting completing a Masters’ Programme specific to Global Health. Seven participants studied an MRes in Global Health, and two undertook a Master of Science (MSc) in Global Health and Global Health and Development, respectively. Additionally, four (11%) participants undertook an MRes in Epidemiology, and two (5%) did an MSc in Control of Infectious Diseases, both key themes within GHE. Eleven (30%) participants reported that their intercalated degree programme involved field research abroad, seven of whom indicated they carried out research in Tanzania, the other three undertook research in one of the Gambia, Sudan, and Zambia.

**Table 3:** Intercalated degree programme subject choice of previous SSC participants

Intercalated degree programme subject	Number
Global Health	9
Epidemiology	4
Control of Infectious Diseases	2
Medical Anthropology	2
Immunobiology	1
Philosophy	1
Reproductive and Sexual Health Research	1
Stem Cells and Regenerative Medicine	1
Not stated	1

When past SSC participants were asked to provide details of any further GHE undertaken elsewhere within the optional medical curriculum, responses mainly fell into two main themes. Firstly, arranging global health electives (five participants), four of whom specified travelling to low and middle-income countries (LMICs) in Sub-Saharan Africa and Southeast Asia, and secondly, attending global health conferences and presentations (three participants). Other optional GHE mentioned included undertaking an SSC in Infectious Diseases (one participant) and tailoring a critical appraisal assignment around a global health topic (one participant).

Four of 35 respondents indicated they had completed or were due to begin postgraduate study related to global health since completion of medical school. Three alumni specified undertaking a DTM&H, one of whom had also completed the RANZCOG Diploma (Diploma of Women’s Health, Royal Australian and New Zealand College of Obstetricians and Gynaecologists), whilst one alumnus was studying an MSc in Public Health. Another 10 participants reported they were considering pursuing a further postgraduate qualification related to global health in the future, again with two specifying the DTM&H.

### Extracurricular activity

Twenty-five (68%) study participants indicated that they were involved with a student global health organisation at a local or national level during medical school. Ten reported involvement with one organisation, 11 with two organisations and four with at least three organisations. The most common organisation students were involved in was Students for Global Health (formerly Medsin) **( Table 4).** This is perhaps the most prominent student global health network in the UK, which as of 2014-2015, was found to be established and active at 28 (85%) of 33 UK medical schools ( Matthews, Davies and Ward, 2020).

**Table 4:** Previous SSC participant involvement with student global health organisations during medical school

Student global health organisation	Number (n=37) n (%)
Students for Global Health (formerly Medsin)	18 (49)
Homed	8 (22)
Students for Kids International Projects (SKIP)	7 (19)
Sexpression:UK	4 (11)
Marrow	3 (8)
Healthy Planet UK	2 (5)
Friends of Médecins Sans Frontières (MSF)	2 (5)
International Federation of Medical Students’ Associations	1 (3)
Kids Action Overseas (Kaos)	1 (3)
Student Action for Refugees: STAR	1 (3)
Student Stop AIDS Campaign	1 (3)
Universities Allied for Essential Medicines (UAEM)	1 (3)

Since completing medical school, 16 (46%) of 35 respondents indicated they were involved with extracurricular activities. Of the 19 participants who reported no involvement with extracurricular activities, eight had just graduated in 2019, and five had graduated in 2018 and were currently undergoing their Foundation Programme Year One. Excluding the 10 participants who had just graduated, a more significant proportion of 14 out of 25 (56%) participants reported extracurricular activity since completing medical school. Such activities included teaching, delivering health-related presentations, being committee members of non-profit organisations, research, mentoring youth and working in volunteer clinics **( Table 5).** Five participants specified details of extracurricular activities related to global health following completion of medical school. These included carrying out leadership roles such as Junior Doctor Sustainability Fellow, Improving Global Health (IGH) Fellow and co-founding the North-East Global Health Network (three participants), as well as mentoring subsequent IGH fellows and MRes Global Health students undergoing research in Tanzania (two participants). Other activities mentioned were volunteer work with refugees (two participants), participation in the Global Health Film Festival (one participant) and work in expedition medicine (one participant).

**Table 5:** Previous SSC participant involvement with extracurricular activities since completing medical school

Extracurricular activity	Number (n=35) n (%)	Number * (n=25) n (%)
Teaching	10 (29)	10 (40)
Health-related presentation	8 (23)	7 (28)
Non-profit committee member	3 (9)	2 (8)
Research	3 (9)	2 (8)
Youth mentorship	2 (6)	2 (8)
Volunteer clinic	1 (3)	1 (4)
Other	2 (6)	2 (8)

* adjusted for participants who had just graduated from medical school.

### International Work

Five (14%) of 35 respondents had worked outside of the UK, whilst 19 (54%) were considering working abroad in the future. Responses regarding previous or planned international work could be categorised as humanitarian work (four participants), work in low resource setting (three participants), international GHE programmes including the IGH programme and Diploma in Tropical Medicine East African Partnership (two and one participants respectively), work with the World Health Organisation (two participants), work in Australia and New Zealand (two participants), expedition medicine (one participant) and research (one participant). Two participants also mentioned living outside the UK before attending medical school and thus returning after completing training.

### Knowledge and Attitudes

Twenty-five (71%) of 35 respondents stated that the Newcastle medical school SSC in Global Health had influenced their clinical practice. Eight (23%) participants felt it did not, and two (6%) were unsure. Table 6 shows common themes identified when participants were asked to describe the most significant influence on their clinical practice.

Sixteen (46%) of 35 respondents believed the SSC played a role in their career decisions, 10 (29%) believed it did not, and nine (26%) were unsure. Table 7 shows common themes identified when participants were asked to describe the most significant influence on their career choice. Although perspectives on the Global Health SSC’s career influence were primarily positive, one participant remarked that the experience highlighted that they did not want to pursue a career in global health despite having an interest in the field.

**Table 6:** Themes identified from responses to the question “Describe the most significant influence of the Newcastle medical school SSC in Global Health on your clinical practice”

Theme	Number (n=22)	Quotes
Fostered critical thinking and awareness of wider determinants of health in a global context	11	“Better understanding [of] global issues.” “Gave me the perspective to be able to observe NHS healthcare in comparison and contrast with the healthcare needs of other countries around the world.” “Helped me to think broadly about the social/cultural/environmental determinants of health, so in clinical practice, I am more holistic in my approach to treating patients.” “I currently work with many people from all across the world. I understand them in their socioeconomic and cultural context far better than many of my peers.” “Knowledge of global health issues and their impact on social determinants of health.” “Made me consider and think about the scope of medicine outside of the NHS and abroad, and the applications of medicine other than in clinical practice.” “Made me more aware of the difficulties faced in global healthcare and how models must be long term and rooted in what the community needs.” “More aware of the wider world around us.” “Opened up [my] perspective on current socioeconomic implications on healthcare, encouraged me to consider varying aspects of the healthcare setting I work at. Enabled me to see what kind of research and clinical work can benefit communities in need.” “Piqued my interest in global health, and introduced me to many novel and useful concepts and techniques within global health.” “Synthesising information and thinking critically about a topic from a range of perspectives.”
Cultural understanding	4	“Greater awareness and understanding, especially when treating migrant populations.“ “I currently work with many people from all across the world. I understand them in their socioeconomic and cultural context far better than many of my peers.” “...I feel better equipped to appreciate and respect cultural differences.” “seeing...how important trying to understand culture is to improve health [and] relevant to communities in the UK.”
Motivation to pursue work abroad	4	“Considering working outside of the UK in further practice.” “Made me consider and think about the scope of medicine outside of the NHS and abroad.” “Made me further consider options of working abroad. Hearing first-hand experience of work abroad and understanding some of the pathways into this.” “Now keen to consider careers in developing countries and outside of [the] UK.”
Motivation to pursue additional global health education/training	4	“Advice from doctors in how to get into working in a global health setting.” “...facilitated MRes with research in Tanzania / inspiration to apply for DTM&H.” “Influenced my preferences in having one of my SHO rotations in Paediatric Infectious Diseases.” “Played part in decision to apply to Masters.”
Importance of maintaining ethical and professional standards	2	“A session on ethical electives most likely influenced my clinical practice during the elective period and meant I was more cautious about not doing anything I would not be comfortable doing in the UK when abroad.” “Listening to how best to improve global health outcomes without crossing ethical or professional boundaries.”

**Table 7:** Themes identified from responses to the question “Describe the most significant influence of the Newcastle medical school SSC in Global Health on your career choice”

Theme	Number (n=16)	Quotes
Knowledge of career paths combining clinical work with engagement in global health	4	“Demonstrat[ed] it’s possible to work in GH alongside a clinical NHS job.” “Demonstrated the different opportunities to get involved with global health as a clinician and that this is possible for multiple specialities, and at more stages in our career than I had previously realised.” “...introduced me to ways of incorporating global health into my career that I hadn’t previously thought of.” “Made me consider GP as a possible speciality to train in - considering the large set of skills GPs need to function in the community and how useful they can be in any setting.”
Motivation to pursue work abroad and in developing countries	4	“I also want to take some periods to work abroad, or in partnerships between NHS trusts and those abroad.” “Now keen to consider careers in developing countries and outside of [the] UK.” “Reinforced my interests in tropical medicine and clinical medicine in developing countries.” “Working abroad.”
Motivation to pursue research in global health	4	“Encouraged me to do an MRes in global health, which lead me to getting an AFP with research in ID/global health. In my future career, I am keen to continue a research component which I don’t think I would have considered previously.” “...GPs can be heavily involved in public health research of a particular community, and [it] made me realise that is an option.” “...influenced my decision to undertake research in Tanzania with a Paediatric cohort.” “Undertaking my AFP in global mental health.”
Awareness of ethical implications of working abroad	2	“made me more aware of the ethical implications of work abroad and factors to consider in respect to this.” “listening to how best to improve global health outcomes without crossing ethical or professional boundaries.”
Commitment to pursuing work with greater public health effect	2	“[Influenced me] to try and pursue a career that will be sustainable and have large public health effect.” “It made me question how big an impact on health being a doctor has... and has made me consider pursuing work in health policy and public health, potentially alongside clinical practice.”

## Discussion

### Principal findings

Of medical students who participated in the SSC in Global Health at Newcastle University between 2011-2017, 71% reported influence on their clinical practice, and 46% noted impact on their career decisions. Whilst a causative relationship cannot be inferred, many alumni have pursued, or are considering, further global health education and extracurricular activity outside of their training programme, as well as international health work, often in underserved communities. Despite this, there was limited interest in Public Health speciality training, consistent with average findings from UK medical graduates in 2015.

### Comparison with prior studies

Previous studies are predominately US-based and focused on the impact of global health experiences abroad. In 2001-2002, a study of participants on the University of Wisconsin IHFP, involving intensive coursework followed by field experience in a developing country, found most fellows experienced a positive impact on their careers ( Ramsey *et al.,* 2004). Four to seven years later, fellows were more likely to engage in primary care specialities than those without fellowship experience, and many went on to work with underserved populations and engage in community health activities ( Ramsey *et al.,* 2004). SSC participants were also more likely to choose a General Practice career over other specialities, though this was in keeping with their UK counterparts **( Table 2).** Similarly, between two and eight years after completion, 46% of SSC alumni had engaged in extracurricular activities, mostly teaching and delivering health-related presentations **( Table 5)**, though several also specified working in humanitarian or low resource settings. These results support previous literature suggesting global health experiences, including electives abroad, can inspire or reinforce student’s motivation to volunteer, work with underserved populations and work abroad ( Godkin and Savageau, 2003; Thompson *et al.,* 2003; Jeffrey *et al.,* 2011; Umoren *et al.,* 2015; Lu *et al.,* 2018).

Several students who undertook the SSC in Global Health later pursued, or were considering, other postgraduate qualifications in public health. Likewise, IHFP fellows were more likely to obtain Master of Public Health (MPH) degrees than their US counterparts ( Ramsey *et al.,* 2004). Despite this interest, only one SSC participant selected public health as their top choice of speciality; however, SSC alumni were comparatively more likely to choose public health training than UK medical graduates (3% versus 0.9%). Similarly, a 1995 survey of 60 Columbia University graduates between 1979-1994 found that those who took an elective course in public health were more likely to specialise in preventive medicine than a comparative group of their counterparts (3% versus <0.5%: P=0.001) ( Rosenberg, 1998). Physicians who studied an MPH were more likely to pursue careers in academic, governmental, and corporate practice settings and spend more time dedicated to non-clinical work ( Rosenberg, 1998). Likewise, a 2007-2009 cohort study at Tulane University showed, compared to those without an MPH, medical graduates with an MPH were more likely to work in academic or government institutions 10-20 years after graduation. The study also showed that MPH holders were 39.84 times (95% CI 12.13-107.38) more likely to practice public health and conduct public health research and produce scholarly works ( Krousel-Wood *et al.,* 2012).

### Strengths and weaknesses of the study

This study contributes a UK perspective to an evolving body of research exploring the impact of global health teaching on medical students regarding their clinical practice and career choice. Understanding this is essential to the case for increased GHE for medical students, contested by increasing time pressures on an already oversubscribed medical curriculum. The General Medical Council (GMC)’s Outcomes for Graduates 2018 gives considerable emphasis to global health knowledge and skills ( General Medical Council., 2018). Therefore, investigating the impact of global health modules is helpful to establish if this method of delivering GHE can remedy current gaps in core curricula by providing students with fundamental global health competencies whilst allowing them to choose further in-depth study.

This study has several limitations. The small sample size means the results are less reliable and must be interpreted with caution. As only 51% of eligible participants responded, the results do not reflect the entire SSC Global Health alumni cohort, and not all global health-related activities were necessarily identified. Furthermore, two of the 37 survey participants did not complete all mandatory questions in the survey, leaving some questions with only 35 respondents. Many respondents were more recent graduates or undergoing the UK Foundation Programme, therefore had had less time to explore further global health opportunities. The questions on the perceived impact on clinical practice and career choice rely on introspective reporting and lack objectivity. There was an inherent risk of reporting bias as those who completed the survey were more likely to have maintained an interest in global health and may have over-reported the positive impacts on their clinical practice and career to favour the inclusion of global health modules in the curriculum.

### Implications for policymakers

Eaton *et al.* ( Eaton, Redmond and Bax, 2011) suggest that whilst the most effective way to deliver GHE is to integrate global health teaching components within mainstream curricular teaching, most medical schools rely on a contributory model of global health content alongside the core curriculum, such as SSCs. Indeed of 26 UK medical school faculty reporting optional global health teaching, 23 reported offering a special module ( Matthews, Davies and Ward, 2020). Whilst we do not propose a causal relationship, an SSC in Global Health may be associated with a positive influence on participants’ clinical practice and has at least a reinforcing effect on career choice to seek further global health opportunities. Notably, several participants reported gaining a better understanding of patients in a global context, including socio-economic and cultural aspects of health, leading to a more holistic approach **( Table 6).** Thus, strengthening existing curricular infrastructure and increasing participation in comprehensive global health modules across UK medical schools can help develop graduates fit for practice in a global workplace, including the UK NHS.

### Future research directions

Further exploration of the influence of GHE on postgraduate clinical practice and career development is required. Participants may be followed up again once they have had longer to develop their careers to gather additional information and provide comparative data for analysis. Although the sample was appropriate to test an initial hypothesis, a more extensive confirmatory study is needed to test the associations identified between global health training and postgraduate clinical practice and career development. A cohort study would be better suited to characterise global health teaching’s causal impact on participants with similar baseline characteristics.

## Conclusion

Global health modules in the medical curriculum may affect positive change in students’ clinical practice and help inform their career choice. As many students may have been interested in global health before choosing the SSC, a causative relationship cannot be inferred. However, Newcastle University Medical School SSC Global Health alumni are likely to pursue further global health opportunities, including carrying out postgraduate degrees and research related to global health and participating in international health work. Medical schools that endeavour to produce graduates motivated to tackle our society’s global health challenges should champion comprehensive global health modules for students.

## Take Home Messages

• Global health modules in the medical curriculum can enhance students’ competency to work in an increasingly globalised workplace.
• Medical student participation in global health modules should be facilitated and encouraged to aid positive career development.
• SSCs in Global Health provide an interim solution for medical schools endeavouring to integrate global health components in oversubscribed mainstream curricular teaching.

## Notes On Contributors

Natasha Matthews, BSc (Hons), MBBS, is an Academic Foundation Programme Doctor at Newcastle University, UK. Her interests include global health education, mental health, elderly health, medical ethics, and law. ORCiD: https://orcid.org/0000-0003-2170-9011

Richard Walker, MD, FRCP, is a Consultant Physician, Honorary Professor of Ageing and International Health, with an interest in healthcare of the elderly, at North Tyneside General Hospital, where he was appointed a consultant in 1995. He has a special interest in Parkinson’s Disease (PD) and is the Clinical lead for Northumbria PD Service. He is involved both locally and nationally with the DeDRoN Research Network. ORCiD: https://orcid.org/0000-0003-3155-122X

## Declarations

Richard Walker leads the SSC Global Health at Newcastle University Medical School. The authors alone are responsible for the content and writing of this article.
