# Supplementary material for: Training tomorrow’s doctors: the impact of a Student Selected Component in Global Health during medical school
Source: MedEdPublish (2016). 2021 Jun 2;10:150. [Version 1] doi: 10.15694/mep.2021.000150.1 (PMC10939561; doi:10.15694/mep.2021.000150.1)
Supplement: Peer review history

# Reviewer report: Reviewer response for version 1

**DOI:** 10.21956/mep.18934.r27081

**Published:** 2021-07-14

**Authors:** Richard Hays

**Affiliations:** 1 James Cook University

This review has been migrated. The reviewer awarded 4 stars out of 5 The most interesting thing about this article is that provides a longitudinal view of how one SSC choice fits into medical training and career development. Student have many options for SSCs and some will select global health. Those that do are probably a self-selected group because they had prior interest and this study shows that several follow this up with research or postgraduate training in public health or related topics. Limitations include small numbers that make statistics relatively meaningless and a lowish (51%) response rate. The findings confirm both the prior interest and the support for that interest from the SSC, supporting the GMC reasoning behind mandating some flexibility and choice within medical school curricula. This is a nice little evaluation that will help guide future SSC development. One minor quibble is that my interpretation is a little more conservative - more medical schools should offer an SSC in global health, rather than making all schools include more global health. the Larger numbers in a longer time frame might merit an additional star, but that is for the next report a few years from now.

Reviewer Expertise:

NA

No decision status is available

# Reviewer report: Reviewer response for version 1

**DOI:** 10.21956/mep.18934.r27082

**Published:** 2021-06-09

**Authors:** Ben Canny

**Affiliations:** 1 Monash University

This review has been migrated. The reviewer awarded 4 stars out of 5 This article present some interesting data regarding the effect of an SSC in Global Health Education on subsequent career choices. The data suggest a positive influence in choices. While the dataset is small, some additional information may have assisted the interpretation of the data. These would include: a sense of the number of students choosing this SSC compared with others, including some sense of the "range" of choice, and the outcome data for Newcastle graduates (as individual schools may produce different outcomes. Finally, caution should be exercised in drawing conclusions about more or less students, given the small population size and the difficulty in statistical comparisons.It is great to see this sort of follow-up study, and it presents data we should reflect on in designing our courses.

Reviewer Expertise:

NA

No decision status is available
